# Investigating the role of *Akkermansia muciniphila* Akk11 in modulating obesity and intestinal dysbiosis: a comparative study of live and pasteurized treatments

**DOI:** 10.3389/fmicb.2025.1638771

**Published:** 2025-11-07

**Authors:** Songhui Feng, Weitao Wang, Xin Zhang, Shimaa Elsayed Helal, Nan Peng, Zhenting Zhang

**Affiliations:** 1 State Key Laboratory of Agricultural Microbiology, Hubei Hongshan Laboratory, College of Life Science and Technology, Huazhong Agricultural University, Wuhan, Hubei, China; 2 The Key Laboratory of Environmental Pollution Monitoring and Disease Control, Ministry of Education, School of Public Health, Guizhou Medical University, Guiyang, Guizhou, China

**Keywords:** *Akkermansia muciniphila* Akk11, obesity, pasteurization, gut microbiota, metabolism

## Abstract

**Introduction:**

Obesity has become a major global health concern and is closely associated with imbalances in gut microbiota and chronic low-grade inflammation. Probiotics have been explored for their potential to prevent or alleviate obesity, especially in the case of *Akkermansia muciniphila*. While the standard strain *A. muciniphila* ATCC BAA-835 has been shown to help reduce obesity, significant functional variations among different strains remain a concern. To address this issue, our research investigated the impact of *A. muciniphila* Akk11 (Akk11), a strain sourced from the feces of healthy infants, in both its live and pasteurized forms on obesity.

**Methods:**

Male C57BL/6J mice were fed a high-fat diet to induce obesity and then treated with either live or pasteurized *A. muciniphila* Akk11. Body weight, adiposity, intestinal histology, gut microbiota composition (via 16S rRNA gene sequencing), and short-chain fatty acids (SCFAs) levels were assessed after the intervention period.

**Results:**

We observed that both forms of Akk11 provided protective benefits in obese mice, as evidenced by reductions in Lee’s index and the area of white adipose tissue. In terms of intestinal health, both live and pasteurized Akk11 notably increased the number of goblet cells in the colon while also significantly improving mucosal integrity and enhancing the expression of tight junction proteins. Notably, 16S rRNA gene sequencing revealed that pasteurized Akk11 altered the gut microbiota composition, with significant differences in the dominant intestinal microbiota. The pasteurized Akk11 group showed a marked increase in the abundance of the *Akkermansia* genus. Additionally, both treatments influenced the levels of short chain fatty acids, though their effects varied. Compared to the control group, both live and pasteurized Akk11 treatments led to higher levels of isobutyric and valeric acids. Furthermore, the live Akk11 significantly boosted propionic acid levels, while the pasteurized Akk11 significantly increased butyric acid levels.

**Discussion:**

These findings indicated that both live and pasteurized Akk11 could serve as promising strategies for alleviating obesity linked to high-fat diets. This research supports the potential use of various *A. muciniphila* preparations as therapeutic options for obesity and related health issues in humans.

## 1 Introduction

Excess weight and obesity are characterized by an unhealthy or excessive buildup of fat that can lead to various health complications (World Health Organization, 2024a). Since 1990, the rate of obesity among adults around the world has more than doubled, and the rate among children and teenagers (aged 5 to 19 years) has increased threefold (Phelps et al., 2024). By 2022, it was estimated that over 1 billion people globally would be living with obesity, with 43% of adults falling into the overweight category (Phelps et al., 2024). Roughly one in eight individuals worldwide may face problems linked to obesity (World Health Organization, 2024b). These conditions are connected to numerous health risks, such as a higher chance of developing type 2 diabetes, non-alcoholic fatty liver disease, breathing difficulties, heart diseases, and other challenges affecting both individuals and society as a whole (Heymsfield et al., 2017; Kivimäki et al., 2022; Valenzuela et al., 2023).

Obesity arises from a complex interplay of various elements, including eating patterns, genetic factors, medications, social influences, and mental health issues (Williams et al., 2015; Lin and Li, 2021). Approaches to managing obesity generally consist of changes in lifestyle, behavioral modifications, drug treatments, and surgical options (Apovian et al., 2015). Although both medication and surgery have received approval, they carry specific risks. Surgical interventions often involve reducing stomach size to decrease food consumption, which may result in complications such as bleeding, rupture at the esophagus–stomach junction, and blood clots (Martini et al., 2019). The weight loss drugs sanctioned by the Food and Drug Administration (FDA) and the European Medicines Agency (EMA) include orlistat, phentermine/topiramate, naltrexone/bupropion, liraglutide, setmelanotide, semaglutide, and tirzepatide. These medications can cause side effects such as gastrointestinal issues, nausea, vomiting, and headaches (Blüher et al., 2023; Chakhtoura et al., 2023).

Recent studies suggest that probiotics can influence the gut microbiome, reduce inflammation, and improve glucose metabolism (Kang et al., 2022; Zhang et al., 2022; Li P. et al., 2023; Lee et al., 2024). Research studies have shown that probiotics have the potential to be a safe and effective alternative to traditional medical and surgical interventions for enhancing metabolic health and managing weight. Probiotics are defined as “live microorganisms which, when administered in adequate amounts, confer a health benefit on the host” (Hill et al., 2014). Specific strains of lactobacilli, including *Lactobacillus fermentum* and *Lactiplantibacillus plantarum*, have been shown to effectively reduce obesity in mice by reducing both body weight and fat levels (Ejtahed et al., 2019). Additionally, the innovative probiotic *Akkermansia muciniphila* (*A. muciniphila*), first identified in 2004, has been linked to weight loss benefits. This bacterium, part of the Verrucomicrobia phylum, thrives on intestinal mucin as its energy source (Derrien et al., 2004) and is found in the human gut as well as in the intestines of mice and other non-primates (Derrien et al., 2008; Karcher et al., 2021). A wealth of research has highlighted *Akkermansia* in metabolic regulation and gut health, positively impacting various conditions such as metabolic disorders (Everard et al., 2013; Zhao et al., 2017; Hänninen et al., 2018), liver injury (Keshavarz Azizi Raftar et al., 2021a), neurological problems (Ou et al., 2020), colitis (Paredes-Sabja et al., 2013; Zhai et al., 2019), and aging (Cerro et al., 2021) in mice.

Administering *A. muciniphila* to mice has been shown to improve metabolic issues and obesity caused by a high-fat diet (Everard et al., 2013; Yoon et al., 2021). Remarkably, this bacterium maintains its effectiveness even after pasteurization (Plovier et al., 2016; Depommier et al., 2020). However, there is currently no comprehensive analysis comparing the results from these two scenarios. Furthermore, the inflammatory complications linked to obesity and the influence of gut microbiota in these processes have not received adequate focus. The majority of studies have predominantly used the standard strain ATCC BAA-835. A genomic comparison of human *Akkermansia* species indicates that various subspecies have distinct host preferences and functional characteristics (Karcher et al., 2021). Therefore, it is essential to explore the effectiveness of non-standard strains. In this study, we examined a discovered strain, Akk11, known for its strong resistance to stress (Wang et al., 2025), and evaluated its effects on the host under various experimental conditions, including both live and pasteurized forms, as well as normal and high-fat diets, while measuring their influence on fat content, inflammation, gut barrier integrity and gut microbial populations.

## 2 Materials and methods

### 2.1 Bacterial strains

Akk11 was supplied by Wecare Probiotics (Suzhou) Co., Ltd. and was isolated from infant feces. Akk11 was cultured anaerobically at 37 °C for approximately 48 h in a mucin-based basal medium, as described previously (Derrien et al., 2004). The cells were collected via centrifugation at 8,000 × *g* and 4 °C and then washed twice with sterile, anoxic phosphate-buffered saline (PBS) before being resuspended in the same solution. Furthermore, a pasteurized bacterial solution was created by applying heat treatment at 70 °C for 30 min in a digital water bath, a method typically used to eradicate microorganisms while preserving the bacteria’s potential immunomodulatory effects (Plovier et al., 2016).

### 2.2 Animal and experimental design

A total of 40 male C57BL/6 mice, aged between 6 and 7 weeks and sourced from the Experimental Animal Center at Huazhong Agricultural University, were obtained. These specific pathogen-free mice were kept in a controlled environment with stable temperature and humidity, with a 12-h light/dark cycle. They were housed in groups of five per cage with unrestricted access to food and water. Prior to the experiment, the mice were maintained on a standard diet for 1 week and then randomly assigned to four groups using stratified randomization based on their body weight. This procedure ensured that the mean starting weight was identical across groups so that subsequent changes in body mass could be attributed solely to diet and/or Akk11 treatment rather than to pre-existing inter-group disparities. The standard diet was categorized into a control group (ND) and a high-fat diet consisting of 60% fat and 20% carbohydrates (D12492, kcal/100 g). The high-fat diet cohort was divided into three categories: the model group (HFD), a group receiving 2 × 10^9^ AFU/day of live Akk11 bacteria (HFD + Akk), and a group administered 2 × 10^9^ AFU/day of pasteurized Akk11 bacteria (HFD + PAkk). Both the model and control groups were treated with the same volume of sterile PBS via gavage.

The study spanned a duration of 5 weeks, during which the weight and weekly food consumption were recorded, alongside the collection of fresh feces from the mice. All 40 male mice remained healthy throughout the intervention; no unexpected deaths or removals occurred. Upon completion, all mice were euthanized via cervical dislocation, and samples of blood, white adipose tissue, the liver, the ileum, and the cecum were obtained. Lee’s index is defined as body weight (g) divided by the square of body length (cm^2^). The Institutional Animal Care and Use Committee of Huazhong Agricultural University approved all experimental procedures (approval No. HZAUMO-2024-0111).

### 2.3 Biochemical analysis

Triglycerides (TG), low-density lipoprotein cholesterol (LDL-C), and high-density lipoprotein cholesterol (HDL-C) were quantified using the triglyceride assay kit (A110-1-1), low-density lipoprotein cholesterol assay kit (A113-1-1), and high-density lipoprotein cholesterol assay kit (A112-1-1) from Nanjing Jiancheng Bioengineering Institute. Both tests were performed in accordance with the instructions provided by the manufacturers.

### 2.4 Histological evaluation

Following a 24-h fixation in 4% paraformaldehyde, samples of colon and white adipose tissue were embedded in paraffin and cut into standard thickness sections. These sections underwent a series of dewaxing and hydration processes. To visualize cellular structures, the sections were stained with hematoxylin and eosin (H&E) (Cardiff et al., 2014). Pathological alterations were assessed using a light microscope (80i, Hitachi, Japan) at 100 × magnification (ND, *n* = 6; HFD, *n* = 9). Additionally, colon cells were stained with alizarin blue-periodic acid–Schiff (AB-PAS), and immunohistochemistry was used to identify the presence of tight junction proteins ZO-1, Muc-2, occludin, and claudin-1 in colon tissue at 200 × magnification (*n* = 5). The dimensions of white adipocytes, the relative proportion of goblet cells for AB-PAS staining, and the areas positive for immunohistochemistry were evaluated using Image Pro Plus 6.0 software for image analysis.

### 2.5 Gas chromatography–mass spectrometry analysis

A total of 100 mg/mL mixed standard stock solutions of 5 SCFAs (acetic acid, propionic acid, butyric acid, isobutyric acid, and valeric acid) and 100 mg/mL caproic acid stock solution were prepared in water and ether, respectively. Five SCFAs and a caproic acid working solution series were both prepared by appropriate dilutions of a standard stock solution. A total of 375 μg/mL of internal standard (IS) solution containing 4-methylvaleric acid was similarly prepared with ether. A ten-point calibration curve was established by adding 220 μL of the working solutions, which contained 200 μL of the six-acid working solution series, 20 μL of the caproic acid working solution series, 100 μL of 15% phosphoric acid, 20 μL of 375 μg/mL IS solution, and 260 μL of ether, with a calibration range from 0.02 to 500 μg/mL (0.02, 0.1, 0.5, 2, 10, 25, 50, 100, 250, and 500 μg/mL). Stock solutions were stored at −20 °C before use, and working solutions were prepared before use.

The clear liquid obtained from the homogenized cecal contents was separated by centrifugation at 12,000 *g* for 10 min to eliminate any particulate matter, and this supernatant was utilized for analyzing short-chain fatty acids (SCFAs). The analysis was conducted using a Thermo Trace 1,310 gas chromatography system (Thermo Fisher Scientific, United States) fitted with an Agilent HP-INNOVAX capillary column (30 m × 0.25 mm inner diameter × 0.25 μm film thickness). The chromatographic settings included a split injection with a volume of 1 μL and a split ratio of 10:1. The injection port temperature was maintained at 250 °C, while the ion source temperature was set at 300 °C. The transmission line temperature was also maintained at 250 °C. The temperature program commenced at 90 °C, increased to 120 °C at a rate of 10 °C/min, then increased to 150 °C at a rate of 5 °C/min, and finally reached 250 °C at a rate of 25 °C/min, where it was held for 2 min. Helium served as the carrier gas with a flow rate of 1.0 mL/min. Detection was carried out using a Thermo ISQ LT mass spectrometer (Thermo Fisher Scientific, United States) with an electron impact ionization (EI) source and selected ion monitoring (SIM) mode, operating at an electron energy of 70 eV.

### 2.6 16S rRNA gene sequencing and data analysis

Cecal samples were promptly frozen in sterile cryovials and kept at −80 °C until the DNA extraction process. Genomic DNA was isolated from cecal contents with a kit supplied by Tiangen Biotech (Beijing) Co., Ltd., Beijing, China. DNA concentration and purity were determined using a NanoDrop 2000 spectrophotometer (Thermo Fisher Scientific, United States). Following the quantification of the extracted DNA, a polymerase chain reaction (PCR) was performed targeting the V4 region of the bacterial 16S rRNA gene with the following primers: forward 5′- GTGCCAGCMGCCGCGGTAA −3′ and reverse 5′- GGACTACHVGGGTWTCTAAT −3′. The resulting PCR products were mixed and purified, followed by procedures including end repair, A-tailing, addition of sequencing linkers, and further purification to finalize the library preparation. The prepared library was quantified using the Qubit and Q-PCR methods. After confirming the library’s quality, sequencing was carried out on the NovaSeq 6,000 platform with a PE250 configuration. Paired-end reads were first merged using FLASH (v1.2.11) and subsequently quality-assessed and filtered with fastp (v0.23.1). Sequences were grouped into amplicon sequence variants (ASV) based on 99% similarity and annotated with the Silva 138.1 database. Alpha diversity metrics of the microbial community were computed using QIIME 2, while beta diversity was analyzed through weighted UniFrac distances and visualized using principal coordinate analysis (PCoA) and heatmaps. Statistical comparisons of microbial community structures between groups were performed using permutational multivariate analysis of variance (PERMANOVA) (999 permutations) and analysis of similarities (ANOSIM). Taxon-specific biomarkers were determined using linear discriminant analysis effect size (LEfSe). Additionally, PICRUSt2 was used to estimate microbial functional abundance.

### 2.7 Statistical analysis

Data are presented as mean values with standard error of the mean (SEM). Comparisons among mouse groups were conducted using a one-way ANOVA, accompanied by Tukey’s *post-hoc* analysis or the unpaired t-test. Statistical analyses were performed using GraphPad Prism version 8.00. A *p*-value of less than 0.05 was deemed statistically significant, indicated by the following symbols: * *p* < 0.05, ** *p* < 0.01, *** *p* < 0.001, and **** *p* < 0.0001.

All four experimental groups (ND, HFD, HFD + Akk, and HFD + PAkk) were included in every statistical model. However, because the normal diet (ND) group differed from the two Akk-supplemented groups in both diet background and treatment, we restricted the *post-hoc* pairwise comparisons to the three high-fat-fed cohorts (HFD vs. HFD + live Akk vs. HFD + pasteurized Akk) and separately compared ND to HFD.

## 3 Results

### 3.1 Akk11 effectively reduces obesity in mice

**Figure 1:**
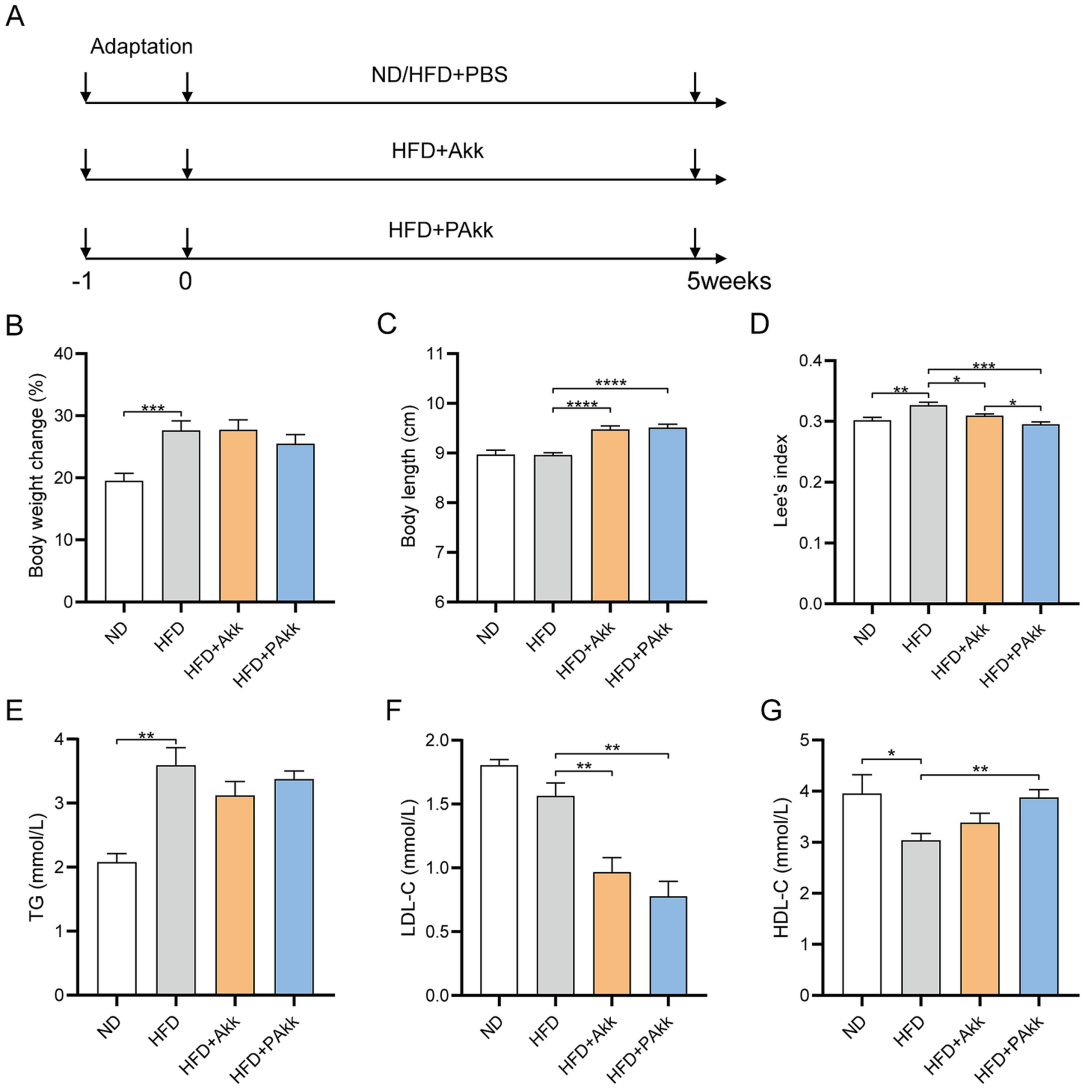
Akk11 improves obesity in HFD mice. **(A)** The experimental design. Effects of Akk11 on **(B)** body weight change; **(C)** body length; **(D)** Lee’s index, defined as body weight (g) divided by the square of body length (cm^2^); **(E)** serum TG; **(F)** serum LDL-C; and **(G)** serum HDL-C. Sample size (body weight change, body length Lee’s index, *n* = 10/ group; serum TG, LDL-C, HDL-C, *n* = 5/ group); * *p* < 0.05, ** *p* < 0.01, *** *p* < 0.001, and **** *p* < 0.0001.

To study the effects of the Akk11 strain on high-fat diet mice, we conducted a 5-week experiment using both live and pasteurized Akk11 for intervention (Figure 1A). It was observed that the addition of Akk11 did not significantly impact weight gain in mice fed a high-fat diet (Figure 1B). Akk11 supplementation did not alter daily food intake compared to the HFD group (Supplementary Table 1). However, statistical analysis revealed that mice supplemented with Akk11 exhibited significantly greater body length than both the high-fat diet and the normal diet groups (Figure 1C). Furthermore, Lee’s index, which provides an objective measure of obesity, showed that all Akk11 supplementations effectively reduced obesity levels, with pasteurized Akk11 demonstrating a stronger effect than its live counterpart (Figure 1D). While a high-fat diet led to elevated triglyceride (TG) levels, neither pasteurized nor live Akk11 supplementation resulted in a significant decrease in TG levels (Figure 1E). Additionally, when compared to the HFD group, both live and pasteurized Akk11 treatments significantly lowered low-density lipoprotein cholesterol (LDL-C) levels (Figure 1F). The high-density lipoprotein cholesterol (HDL-C) levels in the HFD + PAkk group were significantly higher than those in the HFD group (Figure 1G).

**Figure 2:**
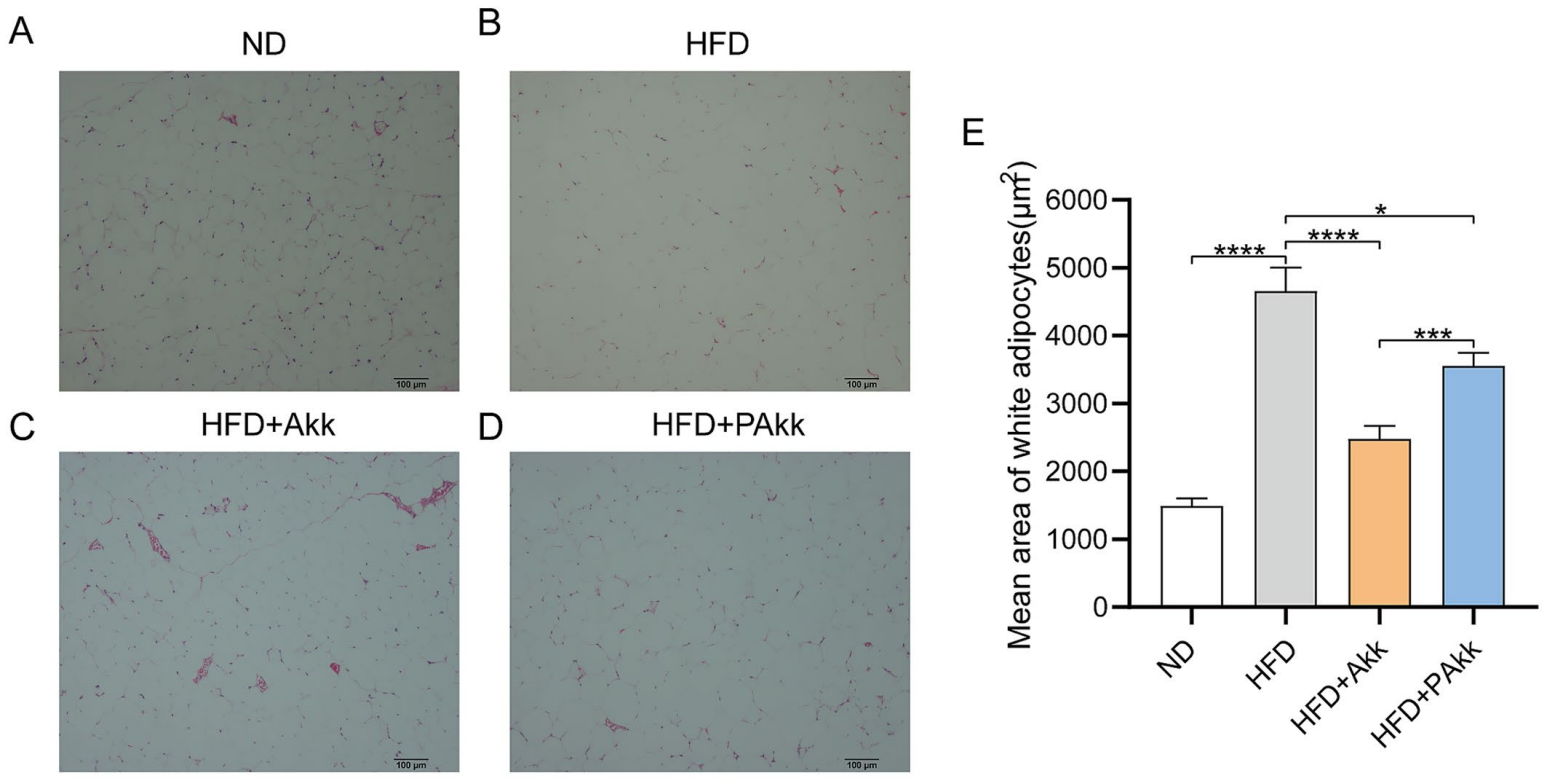
Akk11 reduces the white adipocyte size in HFD mice. **(A–D)** White adipose tissue section of mouse abdomen (HE staining) and **(E)** white adipocyte size. Sample size (ND, *n* = 6; HFD, *n* = 9); * *p* < 0.05, ** *p* < 0.01, *** *p* < 0.001, and **** *p* < 0.0001.

White adipose tissue is essential for maintaining energy balance by storing surplus energy as triglycerides and releasing it when necessary. When white fat cells malfunction, it can lead to metabolic issues such as obesity and diabetes. In our study, we assessed the size of white adipocytes in mice and conducted statistical analyses to determine the effect of Akk11 on adipose tissue. The findings indicated that mice fed a high-fat diet exhibited a notably larger area of white adipocytes than those on a normal diet (Figures 2A,B). However, treatment with Akk11 significantly decreased the white adipocyte area in the high-fat diet group (Figures 2A–E). Following administration of live bacteria, the white adipose tissue area decreased from 4,659 to 2,483 μm^2^, while in the PAkk group, it decreased from 4,659 to 3,559 μm^2^ compared to the high-fat diet group. These findings suggested that both live and pasteurized Akk11 treatments effectively diminish the area of white adipose tissue, thus helping to mitigate obesity.

### 3.2 Akk11 maintains intestinal integrity in HFD mice

**Figure 3:**
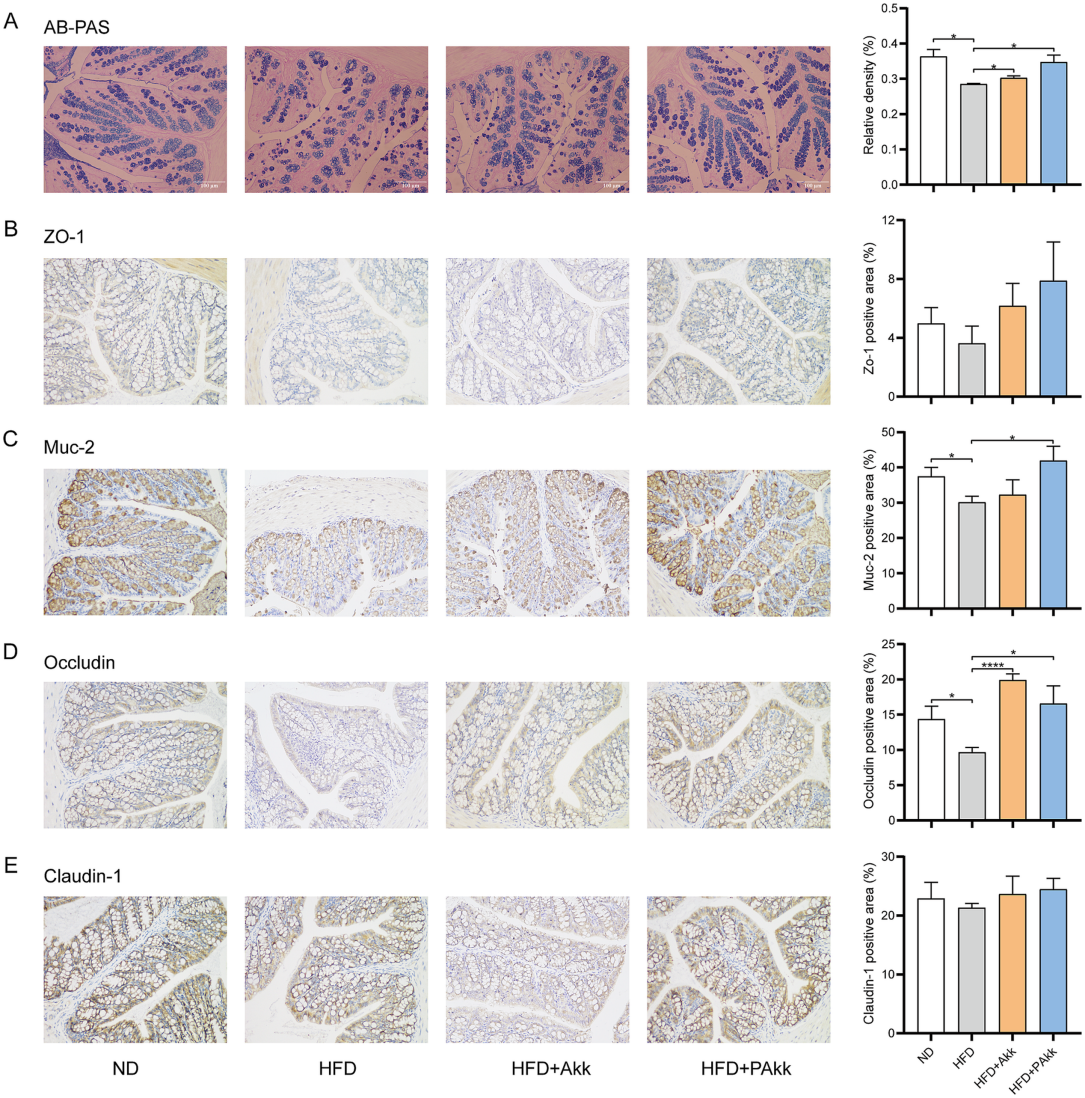
Akk11 enhances the gut barrier. Effects of Akk11 on **(A)** the relative proportion of goblet cells for AB-PAS staining of the mouse colon; **(B)** ZO-1-positive area in the colon; **(C)** Muc-2-positive area in the colon; **(D)** Occludin-positive area in the colon; and **(E)** claudin-1-positive area in the colon. Sample size (*n* = 5/ group); * *p* < 0.05, ** *p* < 0.01, *** *p* < 0.001, and **** *p* < 0.0001.

To assess the impact of live and pasteurized Akk11 treatments on intestinal health, we first measured the number of goblet cells in these groups. AB-PAS staining of the colon revealed that a high-fat diet led to a reduction in the number of goblet cells in the colonic mucosa, whereas live Akk11 showed significant improvement, with pasteurized Akk11 demonstrating even greater effectiveness (Figure 3A).

A high-fat diet usually leads to the disruption of the intestinal barrier. Immunohistochemical analysis revealed that such a diet markedly reduced the levels of tight junction proteins Muc-2 and occludin but did not significantly affect the expression of claudin-1 (Figures 3C–E). After supplementing with pasteurized Akk11, the expression levels of Muc-2 and occludin returned to normal in the colon (Figures 3C,D), and that of ZO-1 also showed a similar trend, although there was no statistical difference (Figure 3B). These findings suggested that pasteurized Akk11 plays a significant role in enhancing intestinal barrier function and helps preserve the integrity of the intestinal mucosa to a certain degree.

### 3.3 Akk11 improves beneficial gut microbiota

**Figure 4:**
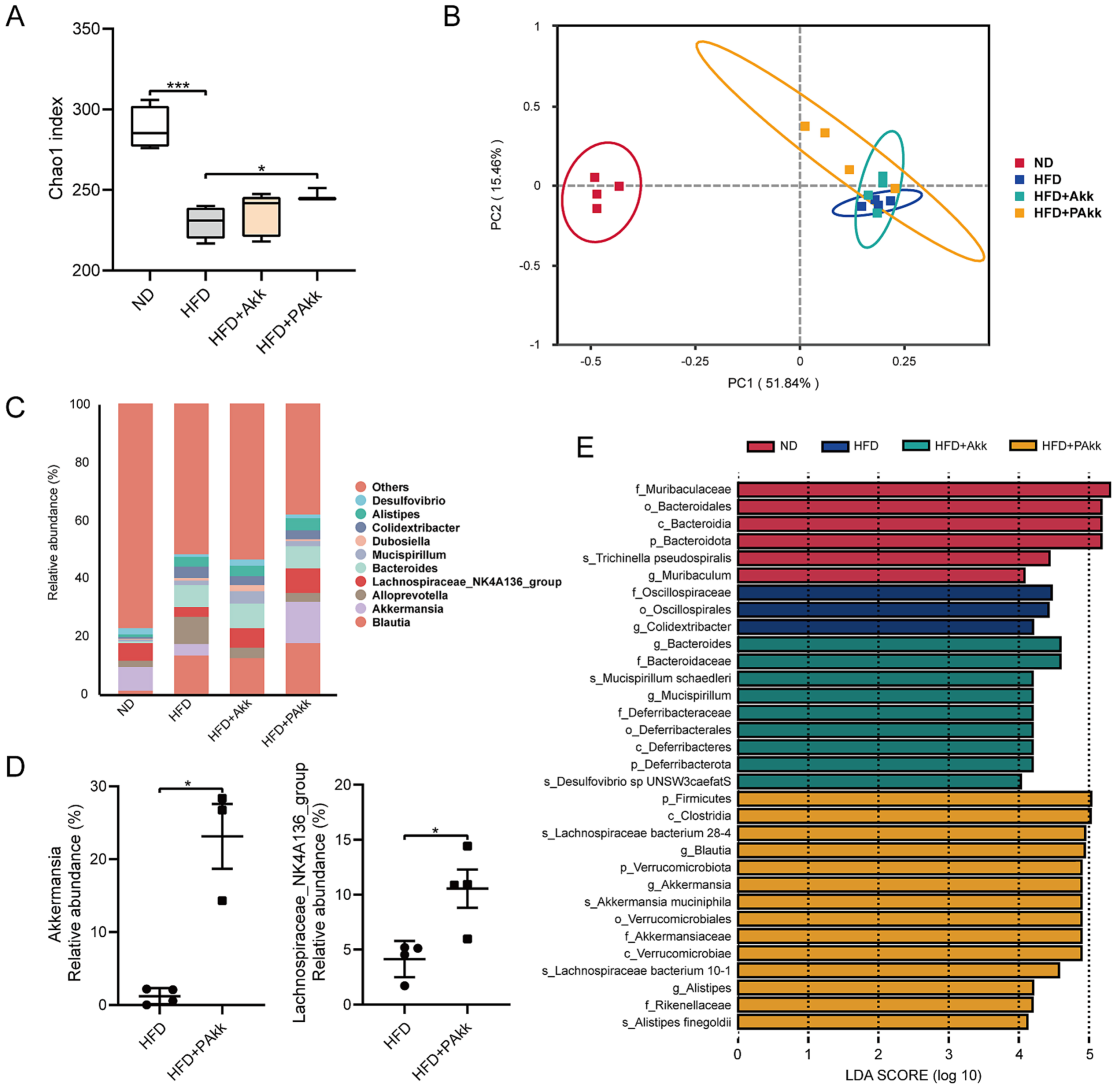
Akk11 alters gut microbiota composition. Effects of Akk11 on the **(A)** *α* diversity index; **(B)** *β*-diversity was determined using Jaccard distance-based principal coordinate analysis (PCoA); **(C)** relative abundance of genus-level gut microbiota; **(D)** comparison of relative abundance of *Akkermansia* and *Lachnospiraceae*_NK4A136_group; and **(E)** LEfSe analysis. Sample size (*n* = 4/ group); * *p* < 0.05, ** *p* < 0.01, *** *p* < 0.001, and **** *p* < 0.0001.

The composition of the microbial community in the cecal contents of mice was notably influenced by dietary changes and treatments with Akk11. The evaluation of the alpha diversity index revealed that a high-fat diet led to a marked decrease in gut microbiota diversity. Although pasteurized Akk11 significantly enhanced the Chao1 index, indicating an increase in microbial richness, this value remained lower than that of the normal diet group (Figure 4A). The structure of the fecal microbiome was examined using PCoA and Jaccard distance analysis. Notably, pasteurized Akk11 resulted in alterations in the beta diversity of the intestinal microbiota under a high-fat diet. Additionally, Bray-Curtis distance analysis of beta diversity showed that the administration of pasteurized Akk11 induced changes in gut microbial beta diversity (ANOSIM, R = 0.248, *p* < 0.05), while no significant differences were observed with live Akk11 (Figure 4B).

Notably, analyses of gut microbial taxonomy revealed a significant increase in the relative abundance of potentially beneficial bacterial groups, such as *Akkermansia* and *Lachnospiraceae*_NK4A136_group, following treatment with pasteurized Akk11 (Figures 4C,D). To explore the unique bacterial community structures across the different treatment groups, linear discriminant analysis effect size (LEfSe) using amplicon sequence variant (ASV) was used to identify the taxa with the greatest difference. In particular, the pasteurized Akk11-treated group showed an increase in the levels of key bacteria, including *Akkermansia*, *Lachnospiraceae,* and *Alistipes finegoldii*, compared to the HFD group. These findings suggest that *Akkermansia*, *Lachnospiraceae,* and *Alistipes_finegoldii* serve as significant biomarkers associated with the supplementation of pasteurized Akk11 (Figure 4E). Overall, these data indicate that pasteurized Akk11 is associated with an increased proportion of potentially health-promoting taxa.

### 3.4 Akk11 elevates the levels of short-chain fatty acids in HFD mice

**Figure 5:**
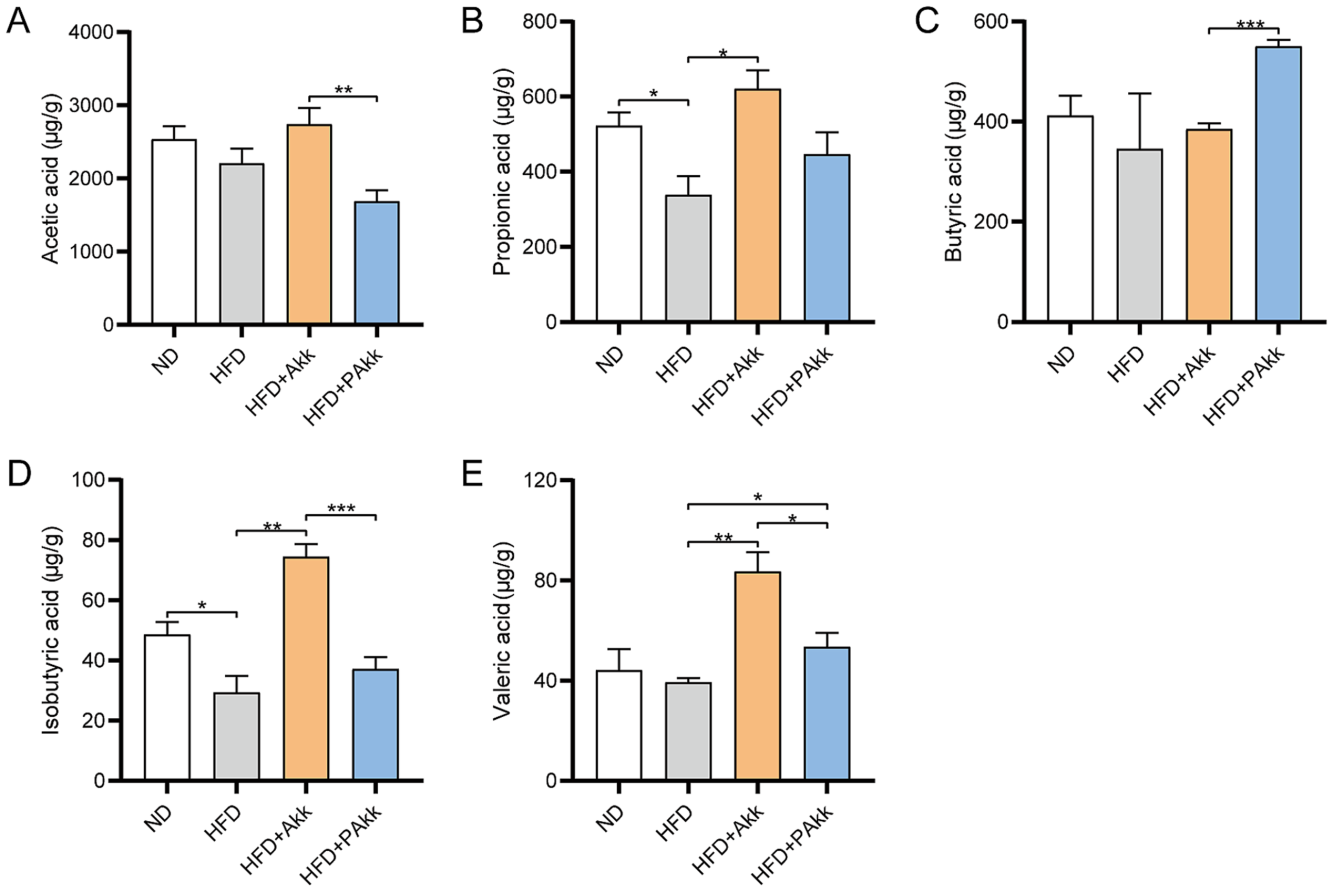
Akk11 increases the content of short-chain fatty acids in the gut of high-fat diet mice. The effect of Akk11 on **(A)** acetic acid; **(B)** propionic acid; **(C)** butyric acid; **(D)** isobutyric acid; and **(E)** valeric acid in the gut of mice. Sample size (*n* = 4/group); * *p* < 0.05, ** *p* < 0.01, *** *p* < 0.001, and **** *p* < 0.0001.

Short-chain fatty acids (SCFAs) are important metabolites that influence gut microbiota. Our findings demonstrated that acetic acid levels were higher in the HFD + Akk group than in the HFD + PAkk group (Figure 5A). The propionic acid, isobutyric acid, and valeric acid levels were increased in the HFD + Akk group when compared to the HFD group (Figures 5B,D,E). However, butyric acid levels increased with the treatment of pasteurized Akk11, and the HFD + PAkk group exhibited significantly elevated butyric acid levels compared to the HFD + Akk group (Figure 5C). Both live and pasteurized Akk11 supplementation led to significant increases in isobutyric acid and valeric acid levels, although the live Akk11 group demonstrated more pronounced effects than the pasteurized variant (Figures 5D,E). These findings suggest that live Akk11 may generate specific SCFAs during its growth in the gut, while pasteurized Akk11 appears to enhance the intestinal environment by influencing bacteria that produce these fatty acids.

### 3.5 Correlation between gut microbiota abundance and physiological indicators

**Figure 6:**
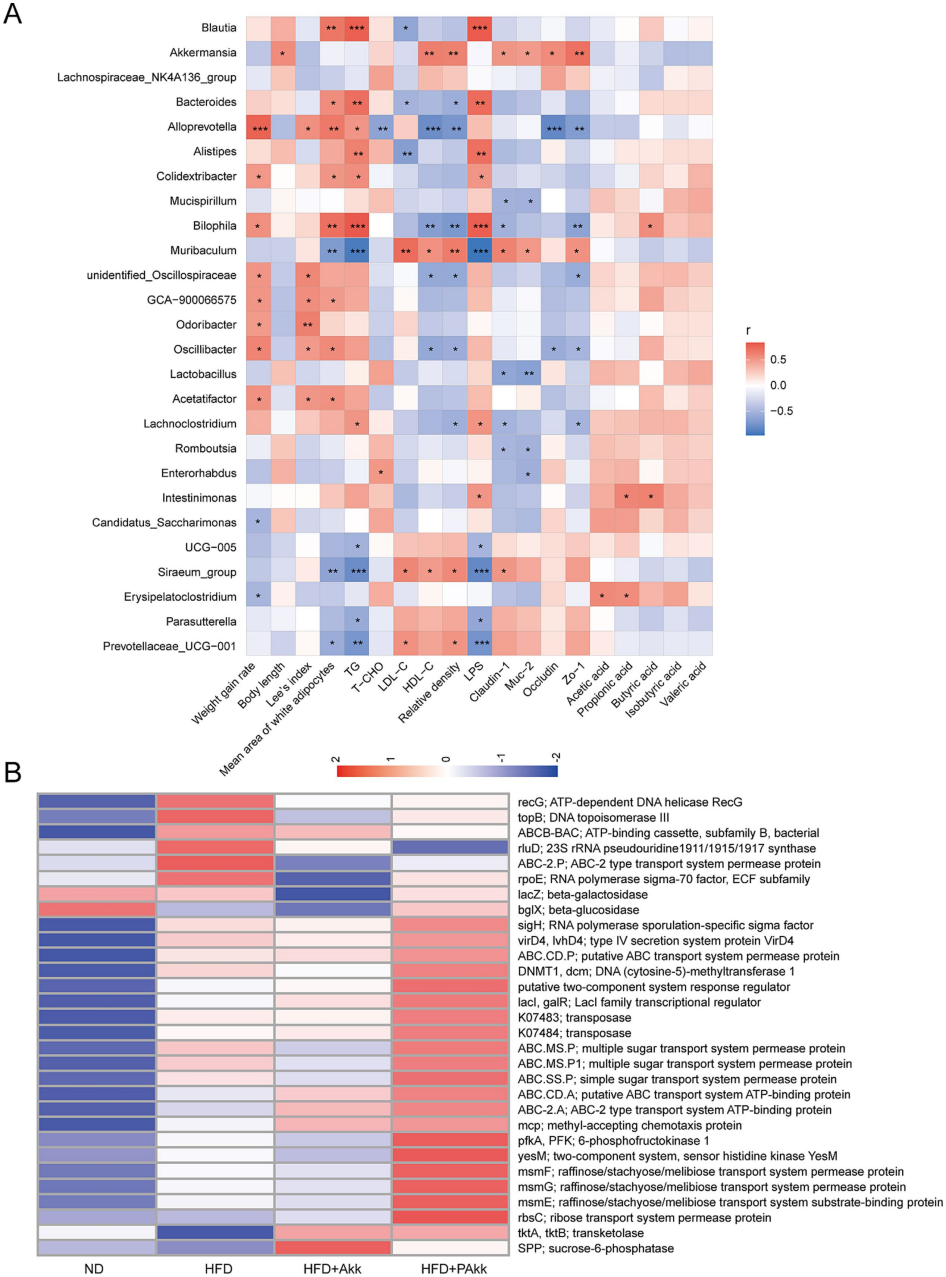
Correlation analysis and prediction of gut microbiota functions. **(A)** Correlation between gut microbiota abundance and physiological indicators. **(B)** The functional gene heatmap. Sample size (*n* = 4/ group); * *p* < 0.05, ** *p* < 0.01.

A correlation heatmap analysis using Spearman’s correlation was performed to examine the correlations among physiological parameters, intestinal SCFAs, and microbial genera in mice fed a high-fat diet. The genera *Muribaculum*, UCG-005, *Siraeum*_group, *Parasutterella,* and *Prevotella ceae* UCG-001 exhibited negative correlations with physiological parameters associated with decreased white adipocyte size, lower serum triglyceride levels, and preservation of the intestinal barrier to mitigate endotoxin effects (Figure 6A). Additionally, *Akkermansia* displayed positive correlations between body length and the integrity of the intestinal barrier (Figure 6A). The presence of *Erysipelatoclostridium* was positively associated with the concentrations of acetic acid, propionic acid, and isovaleric acid. *Intestinimonas* showed a positive relationship between propionic acids and butyric acids, while *Bilophila* was positively associated with butyric acid levels (Figure 6A). Functional predictions of gut microbiota through PICRUSt2 indicated that the high-fat diet group had a generally elevated abundance of functional genes associated with transport compared to those on a normal diet. Notably, pasteurized Akk11 treatment led to an even more pronounced increase in these functional genes (Figure 6B).

## 4 Discussion

The goal of our research was to validate the distinct effects of both live and pasteurized Akk11 strains on obesity, using obese mice as test subjects. Findings based on Lee’s index demonstrated the significant influence of Akk11 (whether live or pasteurized) in reversing high-fat-diet-induced obesity, without affecting their food consumption. Notably, the pasteurized form of Akk11 exhibited a stronger effect in limiting weight gain than its live counterpart. While the metabolic benefits of both live and pasteurized *A. muciniphila* have been extensively documented, the majority of studies have focused on the *A. muciniphila* ATCC BAA-835 strain. Moreover, few have directly compared the live and pasteurized forms within the same experimental setup (Everard et al., 2013; Plovier et al., 2016; Zhao et al., 2017; Yoon et al., 2021).

Additionally, new outcomes were presented—the supplementation of a high-fat diet with Akk11 could augment mouse body length. Notably, both live and pasteurized forms of Akk11 consistently influenced this measurement. Earlier research on animals has shown that *A. muciniphila* positively impacts skeletal health (Keshavarz Azizi Raftar et al., 2021b; Liu et al., 2021; Lyu et al., 2024). Liu et al. supported these results in mice with osteoporosis induced by ovariectomy (OVX), revealing that extracellular vesicles from *A. muciniphila* are crucial for reducing bone deterioration (Liu et al., 2021). In contrast, Lawenius et al. found that pasteurized *A. muciniphila* did not prevent bone loss in the same OVX model (Lawenius et al., 2020). Our study demonstrates that both live and pasteurized Akk11 significantly promote body length, implying that Akk11 may facilitate skeletal growth through different mechanisms.

LDL-C is recognized for its role in transporting cholesterol from the liver to peripheral tissues (Zhang et al., 2021), with elevated levels in the blood linked to a higher risk of atherosclerotic cardiovascular diseases (ASCVD) (Ference et al., 2017). In contrast, HDL-C carries cholesterol from various body regions back to the liver for processing and elimination, thus lowering the chances of atherosclerosis (Feingold, 2022). Our findings indicated a notable decrease in LDL-C levels in both the HFD + Akk group and HFD + PAkk groups, while HDL-C levels increased in the HFD + PAkk group. Both live and pasteurized Akk11 effectively restored the diminished goblet cell count and glycoprotein levels in the colon mucosa due to a high-fat diet. Importantly, pasteurized Akk11 showed a more substantial enhancement of tight junction proteins. Previous research has indicated that live *A. muciniphila* bacteria can thicken the intestinal mucus layer (Everard et al., 2013; Shin et al., 2014). However, our study suggests that although live Akk11 promotes intestinal health, pasteurized Akk11 exerts a more pronounced restorative effect on the intestinal barrier.

A high-fat diet disrupts the balance of intestinal microbes, leading to an increased abundance of specific bacteria such as *Colidextribacter* and *Blautia*. The rise of *Colidextribacter* (Bacillota) associated with a high-fat diet has been connected to Crohn’s disease (Liu et al., 2024). In the HFD + Akk group, there was a notable increase in the levels of Bacteroides (Bacteroidota), *Mucispirillum schaedleri* (Deferribacterota), and *Desulfovibrio*_sp_UNSW3caefatS (Desulfobacterota). SCFAs produced in the intestines are crucial for maintaining the intestinal barrier, regulating energy metabolism, and exerting anti-inflammatory effects (Tong and Lei, 2022). Interestingly, we observed that supplementation with live Akk11 significantly elevated cecal propionic acid levels. Propionic acid is one of the main SCFAs produced by *A. muciniphila* via its mucin-degrading pathway (Derrien et al., 2004). Beyond serving as an energy substrate for colonocytes, propionate can activate GPR43 on intestinal macrophages and regulatory T cells, thereby inhibiting NF-κB signaling and downregulating pro-inflammatory cytokines such as TNF-*α* and IL-6 (Smith et al., 2013). Shin et al. highlighted *Bacteroides* as a key producer of SCFAs (such as succinate, acetate, butyrate, and occasionally propionate) (Shin et al., 2024), and in this study, the enrichment of this genus in the intestines of mice treated with either live or pasteurized Akk11 likely explains the elevated levels of SCFAs following Akk11 supplementation. Additionally, in the HFD + PAkk group, there was an increase in *Lachnospiraceae bacterium* 28_4 (Bacillota), *Akkermansia muciniphila* (Verrucomicrobiota), *Lachnospiraceae bacterium* 10-1 (Bacillota), and *Alistipes finegoldii* (Bacteroidota), with *Lachnospiraceae* bacteria 28-4 being particularly rich in enzymes for butyrate metabolism (Li Z. et al., 2023). The abundance of Akkermansia and Lachnospiraceae NK4A136_group increased with the addition of pasteurized Akk11, and genes related to microbial transport functions were also significantly enriched. Ma et al. noted that Lachnospiraceae NK4A136_group is linked to enhanced intestinal barrier function and obesity traits (Ma et al., 2020). These findings suggest that the beneficial effects of Akk11 on obese mice may be due to the promotion of advantageous gut microbiota. Although elevated *A. muciniphila* abundance in the gut has generally been associated with metabolic benefits, emerging evidence suggests that high *A. muciniphila* levels may also correlate with certain pathological states. Notably, patients with anorexia nervosa (AN) exhibit significantly higher *A. muciniphila* loads than healthy controls (Kipper et al., 2025). Moreover, individuals exhibiting high baseline abundances of *A. muciniphila* and *Bacteroides acidifaciens* are predisposed to increased tumorigenesis in the AOM/DSS colitis-associated cancer model (Achasova et al., 2025). Following the supplementation of Akk11, only the HFD + PAkk group showed a significant increase in *A. muciniphila*, while the HFD + Akk group had the lowest levels across all treatment groups. This difference may indicate niche competition between the introduced live Akk11 strain and the existing *Akkermansia* population, with heat-inactivated Akk11 avoiding such competition while improving the gut environment, leading to an overall increase in *Akkermansia* levels. Consistent with various studies, different *Akkermansia* strains—whether classified as subspecies or lineages—exhibit mutual exclusion when cohabiting the same host (Karcher et al., 2021; Hong et al., 2025).

In summary, this research underscored the ability of Akk11 to alleviate obesity in mice by limiting weight gain, lowering Lee’s index, reducing low-density lipoprotein levels, and decreasing the size of white adipose tissue. These findings suggest that Akk11 serves as a significant contributor to obesity management. Additionally, a notable enhancement in gut microbiota composition was observed, which is increasingly acknowledged as crucial for metabolic health. Specifically, live Akk11 led to a marked increase in short-chain fatty acids, aligning with the bacteria’s role in supporting gut health and energy metabolism. This evidence further emphasizes the importance of gut microbiota in metabolic regulation, as well as the impacts of probiotics and postbiotics on weight control and overall metabolic wellness. Limitations of the present study should be acknowledged. First, all evidence was obtained from a mouse model; equivalent efficacy and safety have not yet been confirmed in humans. Second, the mechanisms by which heat-inactivated Akk11 (postbiotic) alters host adiposity remain unclear, and the specific bioactive components responsible for the observed metabolic benefits were not identified. Third, the intervention period was relatively short, so the long-term consequences of continuous or intermittent Akk11 administration are unknown. Finally, owing to occasional tissue loss and limited DNA/RNA yields, several downstream analyses were conducted on smaller subsets than the primary endpoints. These reduced sample sizes decrease statistical power and widen confidence intervals; thus, the corresponding findings should be viewed as exploratory and require confirmation in larger, independent cohorts. Future studies should investigate the long-term impacts of Akk11 on weight management and clarify its mechanisms, especially in relation to various dietary contexts.

## Data Availability

The datasets presented in this study can be found in online repositories. The raw sequence data are available in the NCBI Sequence Read Archive (SRA) under accession number PRJNA1187494. All other data supporting the findings of this study are provided within the article and its supplementary material.
