# Supplementary material for: Investigating the role of Akkermansia muciniphila Akk11 in modulating obesity and intestinal dysbiosis: a comparative study of live and pasteurized treatments
Source: Front Microbiol. 2025 Nov 7;16:1638771. doi: 10.3389/fmicb.2025.1638771 (PMC12634622; doi:10.3389/fmicb.2025.1638771)
Supplement: Supplementary file 1 [file Table_1.DOCX]

Supplementary Material

# Supplementary Table

# Supplementary Table 1. Weekly Dietary Intake in Mice (g).

| week | ND | HFD | HFD+Akk | HFD+PAkk |
| --- | --- | --- | --- | --- |
| 1 | 89.33±5.5 | 76.00±0.6 | 69.57±2.6 | 77.07±3.7 |
| 2 | 100.1±1.2 | 67.90±1.1 | 69.93±1.0 | 68.13±4.6 |
| 3 | 105.7±1.0 | 65.93±2.1 | 69.10±2.6 | 74.33±13 |
| 4 | 100.2±2.6 | 66.70±0.9 | 68.30±4.1 | 64.27±3.6 |
| 5 | 100.1±1.3 | 67.63±1.1 | 73.67±2.5 | 62.37±2.3 |

# Supplementary Figures

**
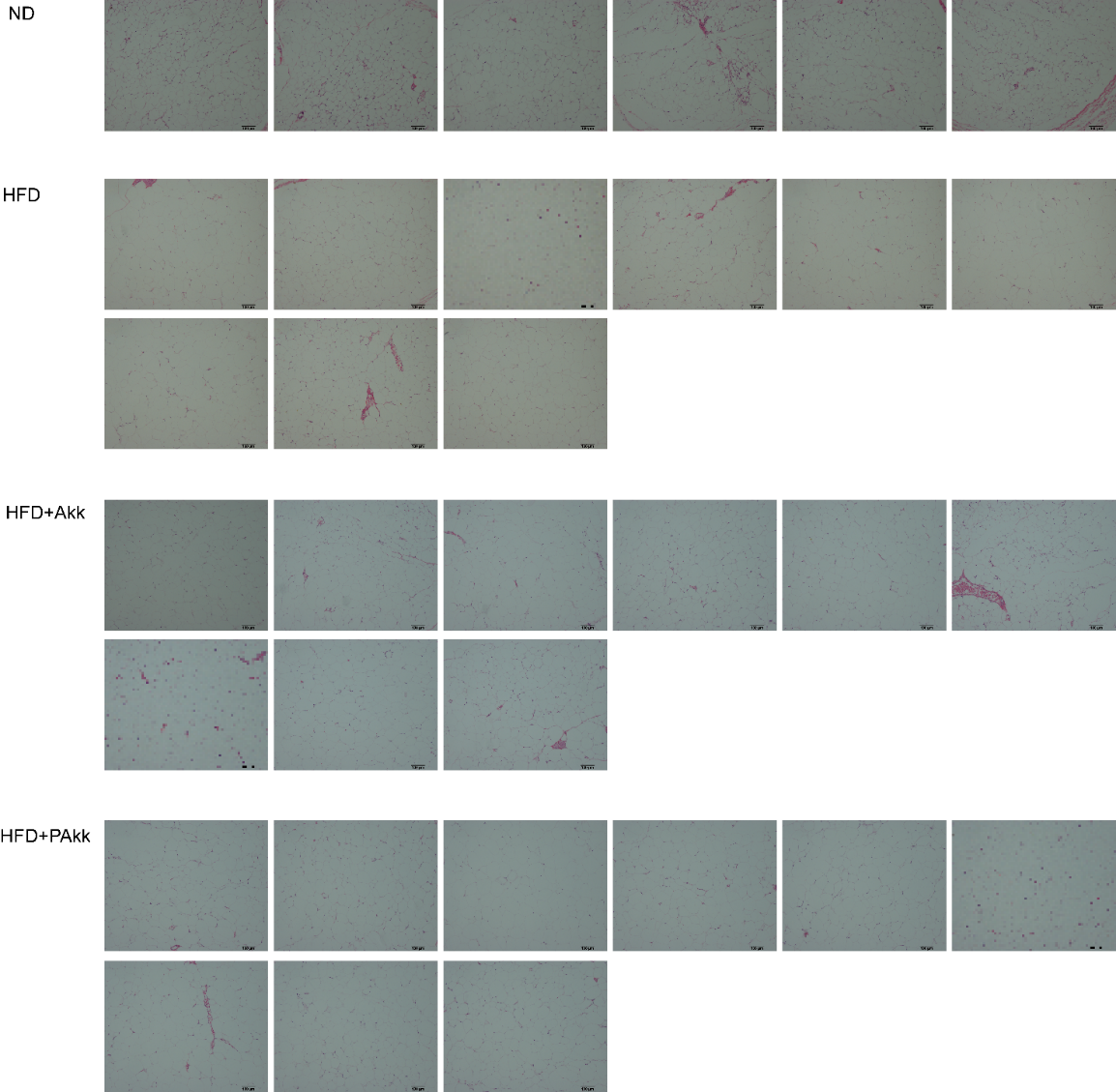
**

**Supplementary Figure 1.** White adipose tissue section of mouse abdomen (HE staining).


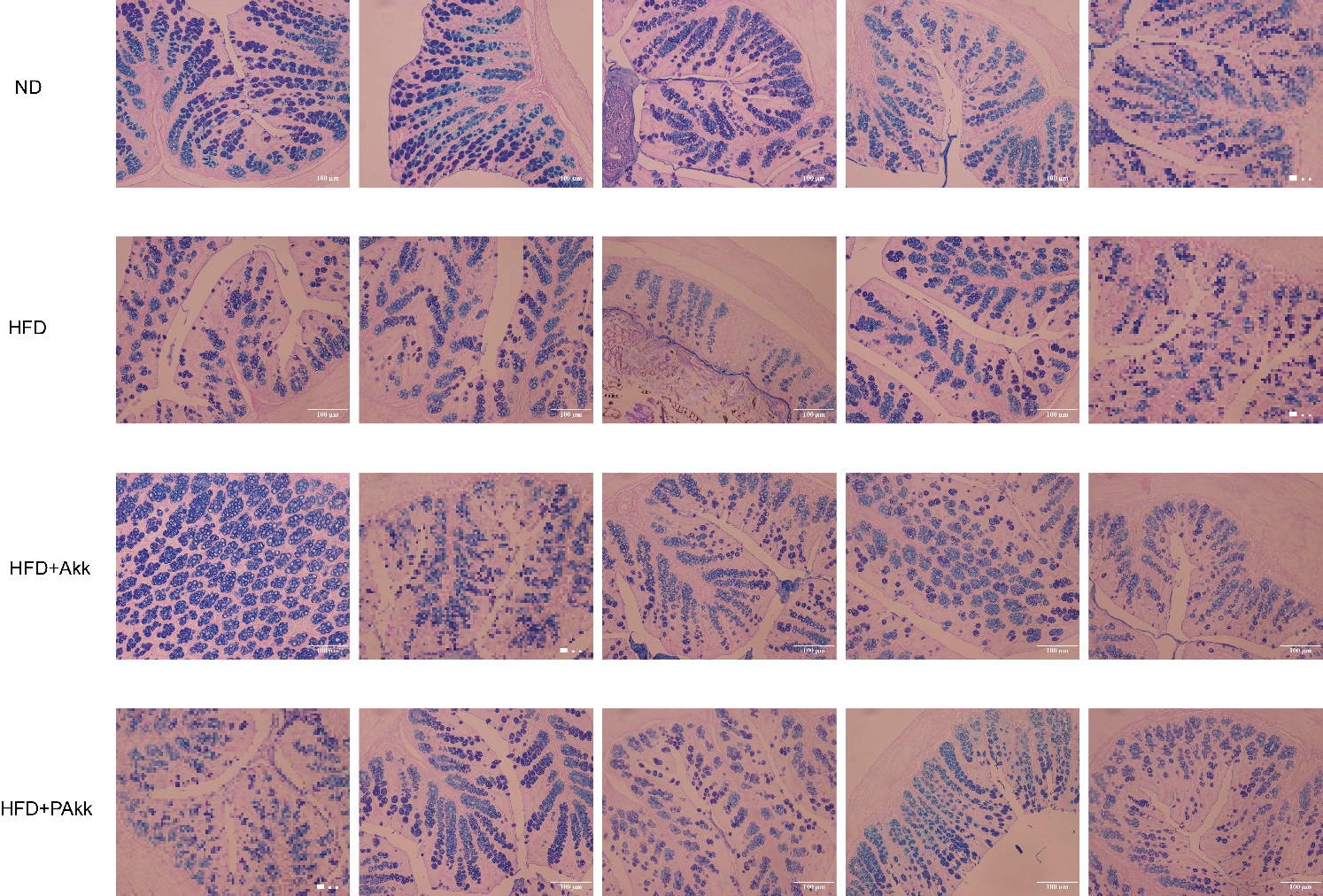


**Supplementary Figure 2.** AB-PAS staining of the mouse colon.


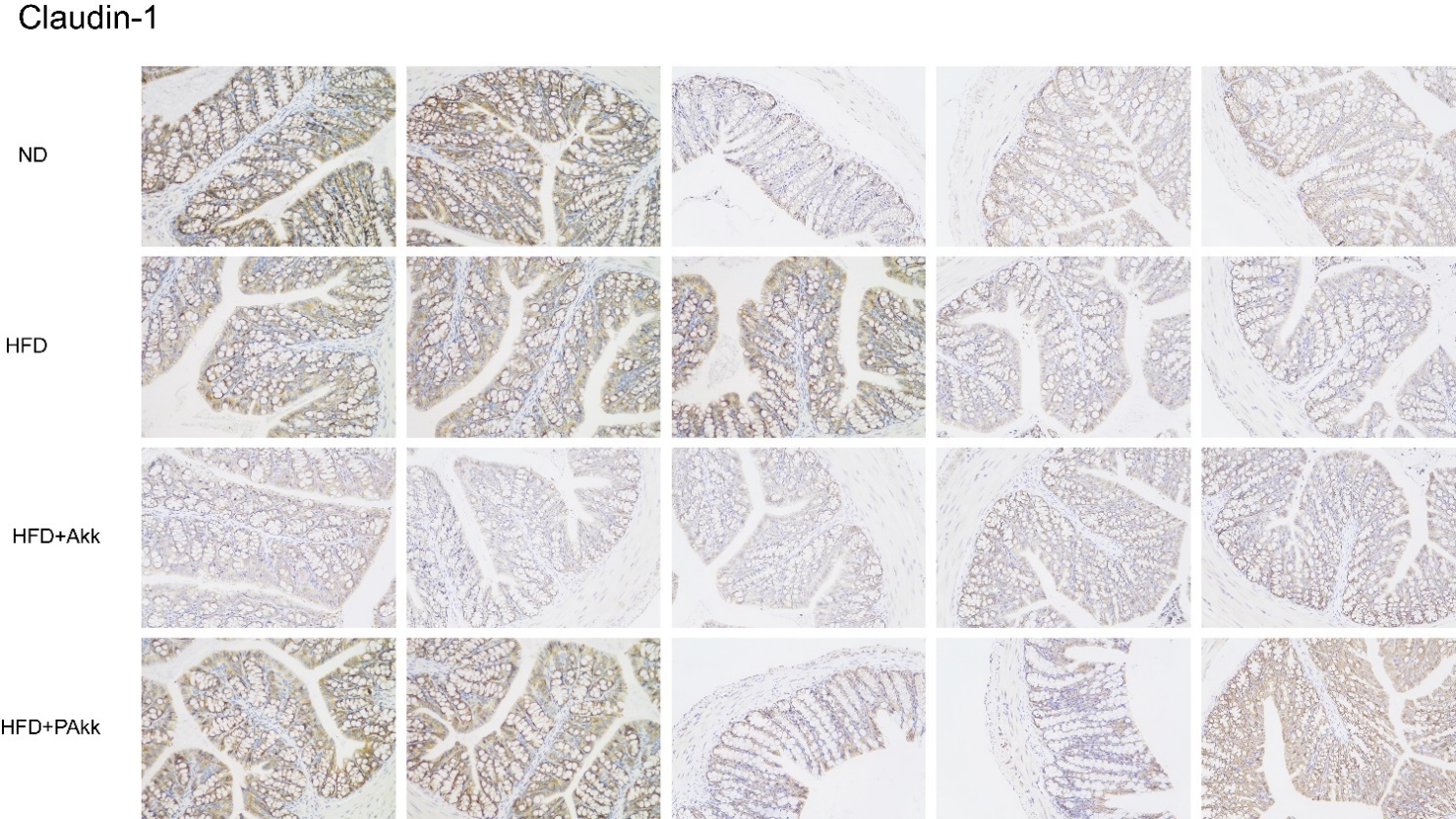


**Supplementary Figure 3.** Claudin-1 immunohistochemical staining of colonic sections.


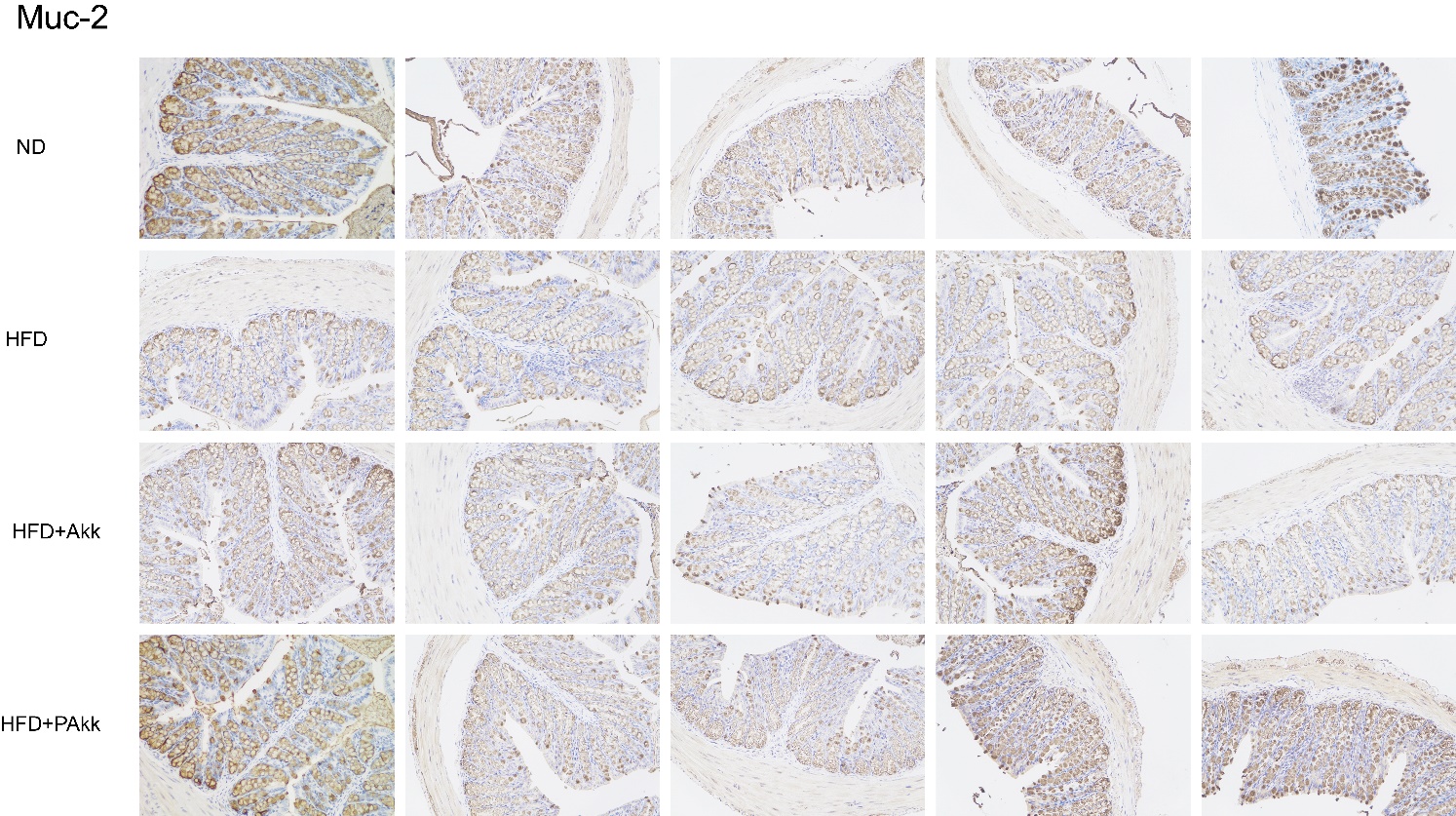


**Supplementary Figure 4.** Muc-2 immunohistochemical staining of colonic sections.

**
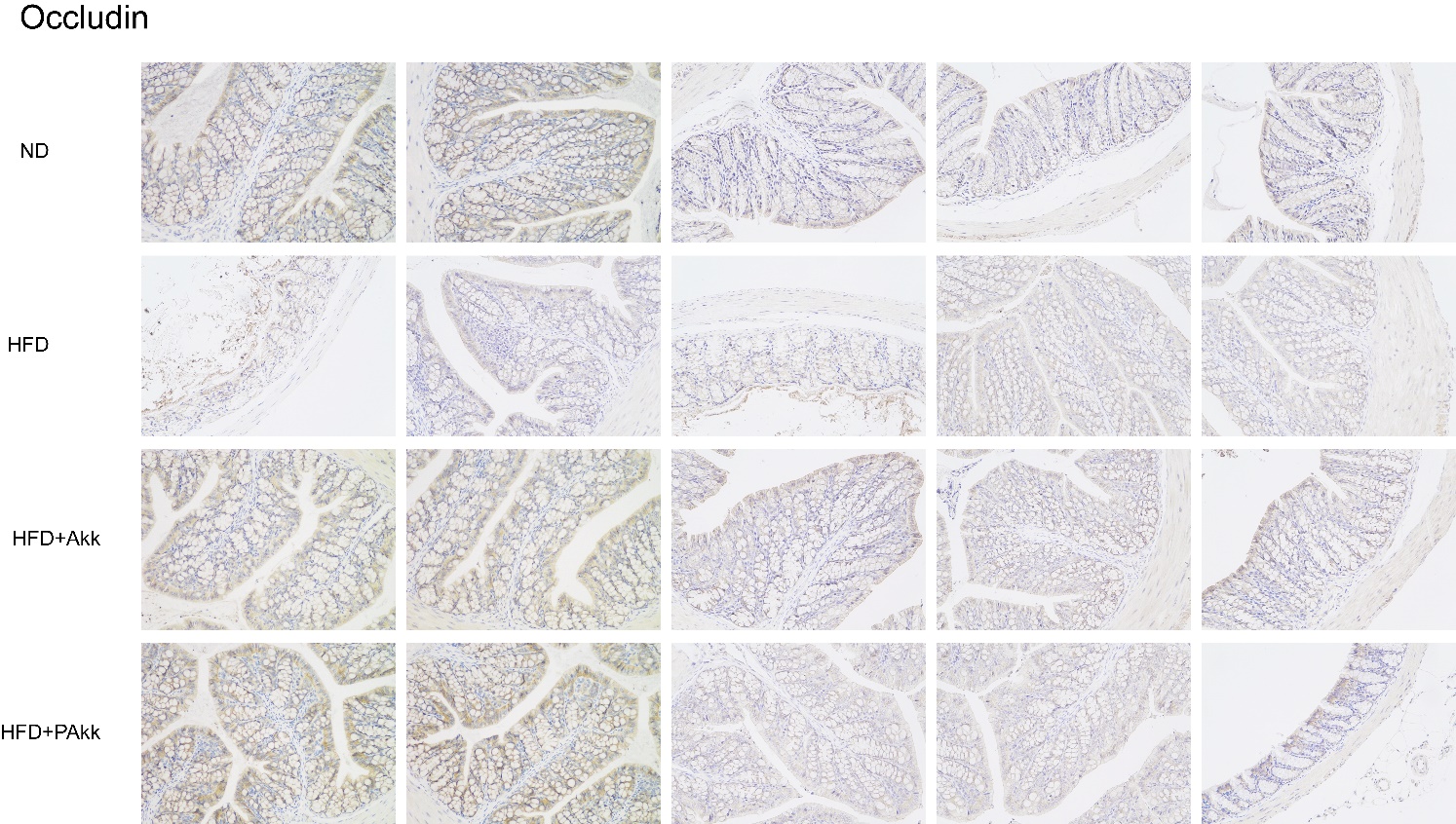
**

**Supplementary Figure 5.** Occludin immunohistochemical staining of colonic sections.

**
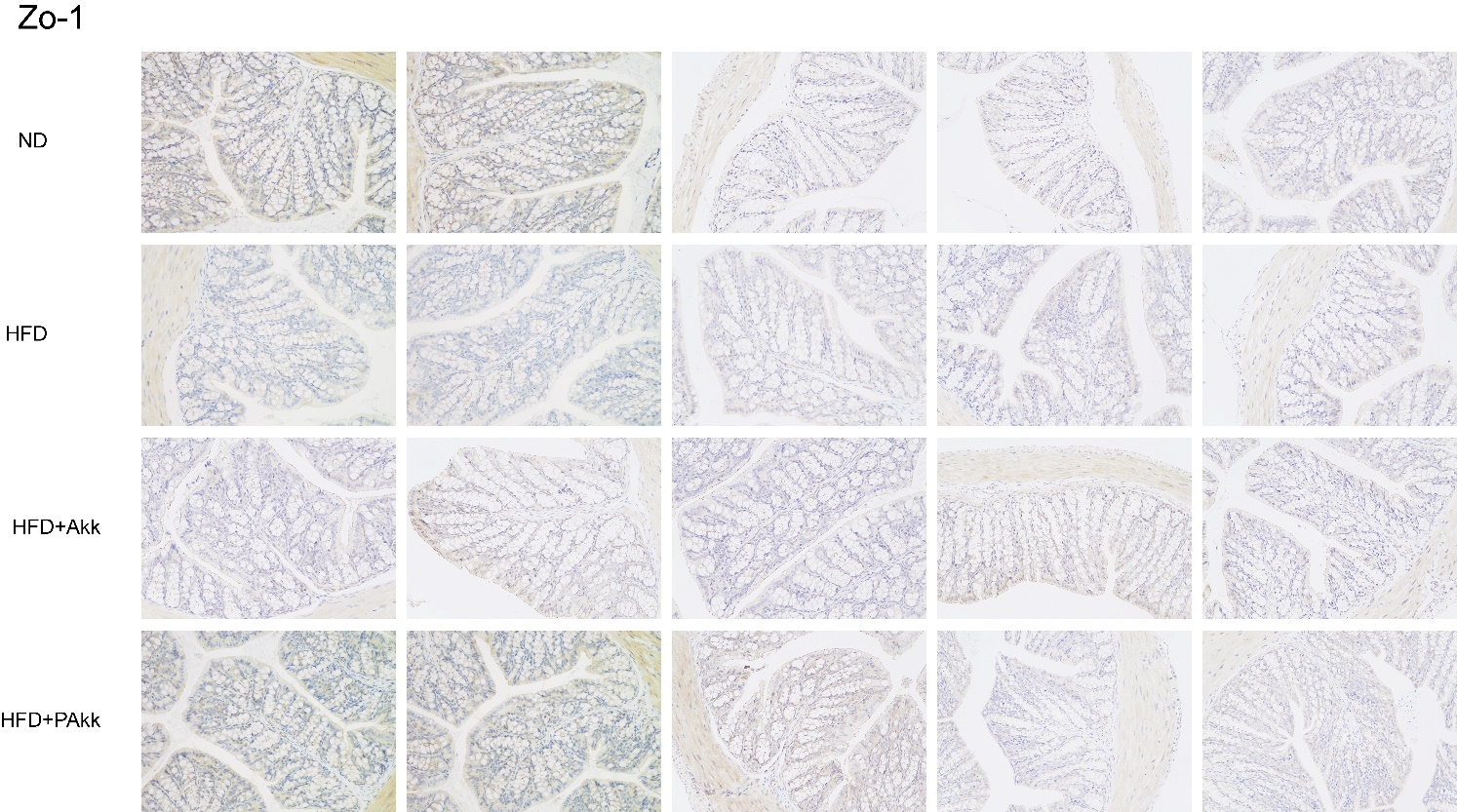
**

**Supplementary Figure 6.** ZO-1 immunohistochemical staining of colonic sections.
